# Supplementary material for: Sublinear scaling of the cellular proteome with ploidy
Source: Nat Commun. 2022 Oct 19;13:6182. doi: 10.1038/s41467-022-33904-7 (PMC9581932; doi:10.1038/s41467-022-33904-7)
Supplement: Supplementary file 1 — Supplementary Information [file 41467_2022_33904_MOESM1_ESM.pdf]

Supplementary Table 1 List of used budding yeast strains

Name	Genotype	Plasmid	Reference
BY4741	*MATa his3Δ1 leu2Δ0 met15Δ0 ura3Δ0*		S288C
BY4742 (YZ945)	*MATα his3Δ1 leu2Δ0 lys2Δ0 ura3Δ0*		S288C
YZ944	*MATa his3Δ1 leu2Δ0 met15Δ0 ura3Δ0 lys2Δ*	PB1650 [*LEU2, URA3, pGal-HO*]	
YZ946i	*MATa/α his3Δ1/his3Δ1 leu2Δ0/leu2Δ0 met15Δ0/MET15 ura3Δ0/ura3Δ0 lys2Δ/lys2Δ*	PB1650 [*LEU2, URA3, pGal-HO*]	
YZ946	*MAT a/a his3Δ1/his3Δ1 leu2Δ0/leu2Δ0 met15Δ0/MET15 ura3Δ0/ura3Δ0 lys2Δ/lys2Δ*	PB1650 [*LEU2, URA3, pGal-HO*]	
YZ947	*MAT α/α his3Δ1/his3Δ1 leu2Δ0/leu2Δ0 met15Δ0/MET15 ura3Δ0/ura3Δ0 lys2Δ/lys2Δ*	PB1650 [*LEU2, URA3, pGal-HO*]	
YZ947i	*MATa/a/α his3Δ1/his3Δ1/his3Δ1 leu2Δ0/leu2Δ0/leu2Δ0 met15Δ0/MET15/MET15 ura3Δ0/ura3Δ0/ura3Δ0 lys2Δ/lys2Δ/lys2Δ*	PB1650 [*LEU2, URA3, pGal-HO*]	
YZ948	*MATa/a/α/α his3Δ1/his3Δ1/his3Δ1/his3Δ1 leu2Δ0/leu2Δ0/leu2Δ0 /leu2Δ0 met15Δ0/MET15/MET15/met15Δ0 ura3Δ0/ura3Δ0/ura3Δ0/ura3Δ0 lys2Δ/lys2Δ/lys2Δ/lys2Δ*	PB1650 [*LEU2, URA3, pGal-HO*]	
YZ949	*MATa/a/a his3Δ1/his3Δ1/his3Δ1 leu2Δ0/leu2Δ0/leu2Δ0 met15Δ0/MET15/MET15 ura3Δ0/ura3Δ0/ura3Δ0 lys2Δ/lys2Δ/lys2Δ*	PB1650 [*LEU2, URA3, pGal*	
YZ950	*MATa/a/a/a his3Δ1/his3Δ1/his3Δ1/his3Δ1 leu2Δ0/leu2Δ0/leu2Δ0 /leu2Δ0 met15Δ0/MET15/MET15/met15Δ0 ura3Δ0/ura3Δ0/ura3Δ0/ura3Δ0 lys2Δ/lys2Δ/lys2Δ/lys2Δ*	PB1650 [*LEU2, URA3, pGal-HO*]	
YZ1375	As YZ944		Used for SILAC
YZ1371	As YZ947		Used for SILAC
YZ1372	As YZ949		Used for SILAC
YZ1373	As YZ950		Used for SILAC
YZ1535	*MATa/leu2Δ/ura3Δ/his3Δ/met15Δ/NQM1::TAP*		
YZ1547	*MATa/leu2Δ/ura3Δ/his3Δ/met15Δ/BRE2::TAP*		
YZ1554	*MATa/leu2Δ/ura3Δ/his3Δ/met15Δ/TUP1::TAP*		

YZ1556	*MATa/leu2Δ/ura3Δ/his3Δ/met15Δ/RTG2::TAP*		
	MATa/leu2Δ/ura3Δ/his3Δ/met15Δ/RFA1::TAP		
YZ1562	*MATa/a/leu2Δ/ leu2Δ/ura3Δ/ ura3Δ/his3Δ/ his3Δ/met15Δ/met15Δ/NQM1::TAP/NQM1::TAP*		
YZ1566	*MATa/a/leu2Δ/ leu2Δ/ura3Δ/ ura3Δ/his3Δ/ his3Δ/met15Δ/met15Δ/BRE2::TAP/BRE2::TAP*		
YZ1602	*MATa/a/leu2Δ/ leu2Δ/ura3Δ/ ura3Δ/his3Δ/ his3Δ/met15Δ/met15Δ/RTG2::TAP/RTG2::TAP*		
YZ1603	*MATa/a/leu2Δ/ leu2Δ/ura3Δ/ ura3Δ/his3Δ/ his3Δ/met15Δ/met15Δ/TUP1::TAP/TUP1::TAP*		
	MATa/a/leu2Δ/ leu2Δ/ura3Δ/ ura3Δ/his3Δ/ his3Δ/met15Δ/met15Δ/RFA1::TAP/RFA1::TAP		
YZ1605	*MATa/a/a/leu2Δ/ leu2Δ/leu2Δ/ura3Δ/ura3Δ/ ura3Δ/his3Δ/ his3Δ/ his3Δ/met15Δ/met15Δ/met15Δ/NQM1::TAP/NQM1::TAP/NQM1::TAP*		
YZ1607	*MATa/a/a/leu2Δ/ leu2Δ/leu2Δ/ura3Δ/ura3Δ/ ura3Δ/his3Δ/ his3Δ/ his3Δ/met15Δ/met15Δ/ met15Δ/BRE2::TAP/BRE2::TAP/BRE2::TAP*		
YZ1617	*MATa/a/a/leu2Δ/ leu2Δ/leu2Δ/ura3Δ/ura3Δ/ ura3Δ/his3Δ/ his3Δ/ his3Δ/met15Δ/met15Δ/ met15Δ/RTG2::TAP/RTG2::TAP/RTG2::TAP*		
YZ1614	*MATa/a/a/leu2Δ/ leu2Δ/leu2Δ/ura3Δ/ura3Δ/ ura3Δ/his3Δ/ his3Δ/ his3Δ/met15Δ/met15Δ/ met15Δ/TUP1::TAP/TUP1::TAP/TUP1::TAP*		
	MATa/a/a/leu2Δ/ leu2Δ/leu2Δ/ura3Δ/ura3Δ/ ura3Δ/his3Δ/ his3Δ/ his3Δ/met15Δ/met15Δ/ met15Δ/ RFA1::TAP/RFA1::TAP/RFA1::TAP		
YZ1606	*MATa/a/a/a/leu2Δ/leu2Δ/leu2Δ/leu2Δ/ura3Δ/ura3Δ/ ura3Δ/ura3Δ/his3Δ/his3Δ/his3Δ/his3Δ/met15Δ/met15Δ/ met15Δ/met15Δ/NQM1::TAP/NQM1::TAP/NQM1::TAP/NQM1::TAP*		
YZ1608	*MATa/a/a/a/leu2Δ/leu2Δ/leu2Δ/leu2Δ/ura3Δ/ura3Δ/ ura3Δ/ura3Δ/his3Δ/his3Δ/his3Δ/his3Δ/met15Δ/met15Δ/ met15Δ/met15Δ/BRE2::TAP/BRE2::TAP/BRE2::TAP/BRE2::TAP*		
YZ1618	*MATa/a/a/a/leu2Δ/leu2Δ/leu2Δ/leu2Δ/ura3Δ/ura3Δ/ ura3Δ/ura3Δ/his3Δ/his3Δ/his3Δ/his3Δ/met15Δ/met15Δ/ met15Δ/met15Δ/RTG2::TAP/RTG2::TAP/RTG2::TAP/RTG2::TAP*		
YZ1615	*MATa/a/a/a/leu2Δ/leu2Δ/leu2Δ/leu2Δ/ura3Δ/ura3Δ/ ura3Δ/ura3Δ/his3Δ/his3Δ/his3Δ/his3Δ/met15Δ/met15Δ/ met15Δ/met15Δ/TUP1::TAP/TUP1::TAP/TUP1::TAP/TUP1::TAP*		
	MATa/a/a/a/leu2Δ/leu2Δ/leu2Δ/leu2Δ/ura3Δ/ura3Δ/ ura3Δ/ura3Δ/his3Δ/his3Δ/his3Δ/his3Δ/met15Δ/met15Δ/ met15Δ/met15Δ/ RFA1::TAP/RFA1::TAP/RFA1::TAP/RFA1::TAP		
	MATa his3Δ/ leu2Δ/ met15Δ/ ura3Δ/tup1::natNT2		
	MATa his3Δ/ leu2Δ/ met15Δ/ ura3Δ/cln3:: natNT2		Euroscarf
	MATa ura3Δ/ leu2Δ/ his3Δ/ met15Δ/ sch9::kanMX4		Euroscarf
	MATa ura3Δ/ leu2Δ/ his3Δ/ met15Δ/ sch9::kanMX4/TUP1-6HA::natNT2		
	MATa ura3Δ/ leu2Δ/ his3Δ/ met15Δ/ TUP1-6HA::natNT2		

Plaasmid	pRS414::sch9as (T492G)		Robbie Loewith

Supplementary Table2 List of used antibodies

Antigen	Name	Host species	Source	Used dilution	Note
Mrp10		Rabbit	Custom-made	1:500	Gift from Prof. Dr. Herrmann
Mrpl40		Rabbit	Custom-made	1:500	Gift from Prof. Dr. Herrmann
Oxa1		Rabbit	Custom-made	1:500	Gift from Prof. Dr. Herrmann
Ilv5		Rabbit	Custom-made	1:500	Gift from Prof. Dr. Herrmann
Mrps5		Rabbit	Custom-made	1:500	Gift from Prof. Dr. Herrmann
Mdh1		Rabbit	Custom-made	1:500	Gift from Prof. Dr. Herrmann
Clb2	Sc-9071	Rabbit	Santa Cruz	1:1000	
Rps23B	Sc-100837	mouse	Santa Cruz	1:500	
Rpl9	AP16409b-ev	Rabbit	ABGENT	1:1000	
Sch9	ab56203	Rabbit	Abcam	1:3000	
PAP	P1291	Rabbit	Sigma - Aldrich	1:1000	Used for all TAP-tagged proteins
Anti-HA-Tag (F-7)	Sc-7392	mouse	Santa Cruz	1:500	
Anti-Puromycin (clone 12D10)	MABE343	mouse	Merck	1:1000	
Rps21	16946-1-AP	Rabbit	Proteintech		
Rps8	Ab201454	Rabbit	Abcam	1:3000	
Rps19	Sc-100836	mouse	Santa Cruz	1:500	
Rpl22	Sc-136413	mouse	Santa Cruz	1:500	
Rpl5	Ab86863	Rabbit	Abcam	1:3000	
Rpl21	Sc-393663	mouse	Santa Cruz	1:500	
Tle1	sc-137098	mouse	Santa Cruz	1:500	
eIF2α	9721S	Rabbit	Cell signaling	1:1000	
p-eIF2α (Ser51)	9722S	Rabbit	Cell signaling	1:1000	
GAPDH (14C10)	2118	Rabbit	Cell signaling	1:1000	

p70 S6 Kinase (49D7)	2708	Rabbit	Cell signaling	1:1000	
Phospho-p70 S6 Kinase (Thr389)	9205	Rabbit	Cell signaling	1:1000	
Goat anti-rabbit HRP		HAF008	R & D	1:5000	
Goat antimouse HRP		HAF007	R & D	1:5000	

Supplementary Table3 List of qPCR primers

Gene	Forward primer (sequence 5'-3')	Reverse primer (sequence 5'-3')
BRE2	GTGTACGGGTATGGTATTAG	TATCACCCTCTTTCAATGGC
RTG2	GCCTCTTATCCAAAGGAGTT	GCGCTCTAGCTCTGTGAGAT
TUP1	CTATCTGACGATTCTGCTGC	GTGGTAGTAGTGGTTGTCATT
ILV5	GGTTACGTTTACCAAACCAC	AGCCAAGAACATACCGTGG
MDH1	CAACTCAGGAAAGGGTTAAC	TCTCTTGTCATCAGACATCAA
MRPL40	GATTCCGTAGTGAGAAACCC	GCTATATCTTTCGCTGTCAAG
RPL9b	GAAACGTCCCAGTTAGAGAT	ATTTTGGGAAACGTCTTCAAC
IRS4	TCTCTACATG AGGGAAATCTG	TGGTTCTGATCAGATTCAGAA
5.8S	GTTGCGGCCATATCTACCAG	AGCACCTGAGTTTCGCGTAT
25S	AGAGCCAATCCTTATCCCG	GGAAGCTCCGTTTCAAAGG
ACT1	CACCCTGTTCTTTTGACTGA	CGTAGAAGGCTGGAACGTTG
FUS1	TTCCAAACCCATAATACCGCA	CACTTTGGGGTCTAACGAAAT
HSP12	AGGTAGAAAAGGATTCGGTGA	GGCCTTGTCAGTGATGTATTC
TMA10	GACAGTCCACGAAGCAAAGT	GCCTTTCCCATAGCCTCCT
NCA3	GATTTGGTGGATGGGCCTCT	GACATTCCAGGTTCACATGC
COX3	TTGAAGCTGTACAACCTACC	CCTGCGATTAAGGCATGATG
18S rRNA	GATGGTAGTCGCCGTGCC	GCCTGCTGCCTTCCTTGG
28S rRNA	AGAGGTAAACGGGTGGGGTC	GGGGTCGGGAGGAACGG
25S rDNA	TACCTTCGGTGCCCGAGTTGTAAT	ACCCTCTATGACGTCCTGTTCCAA

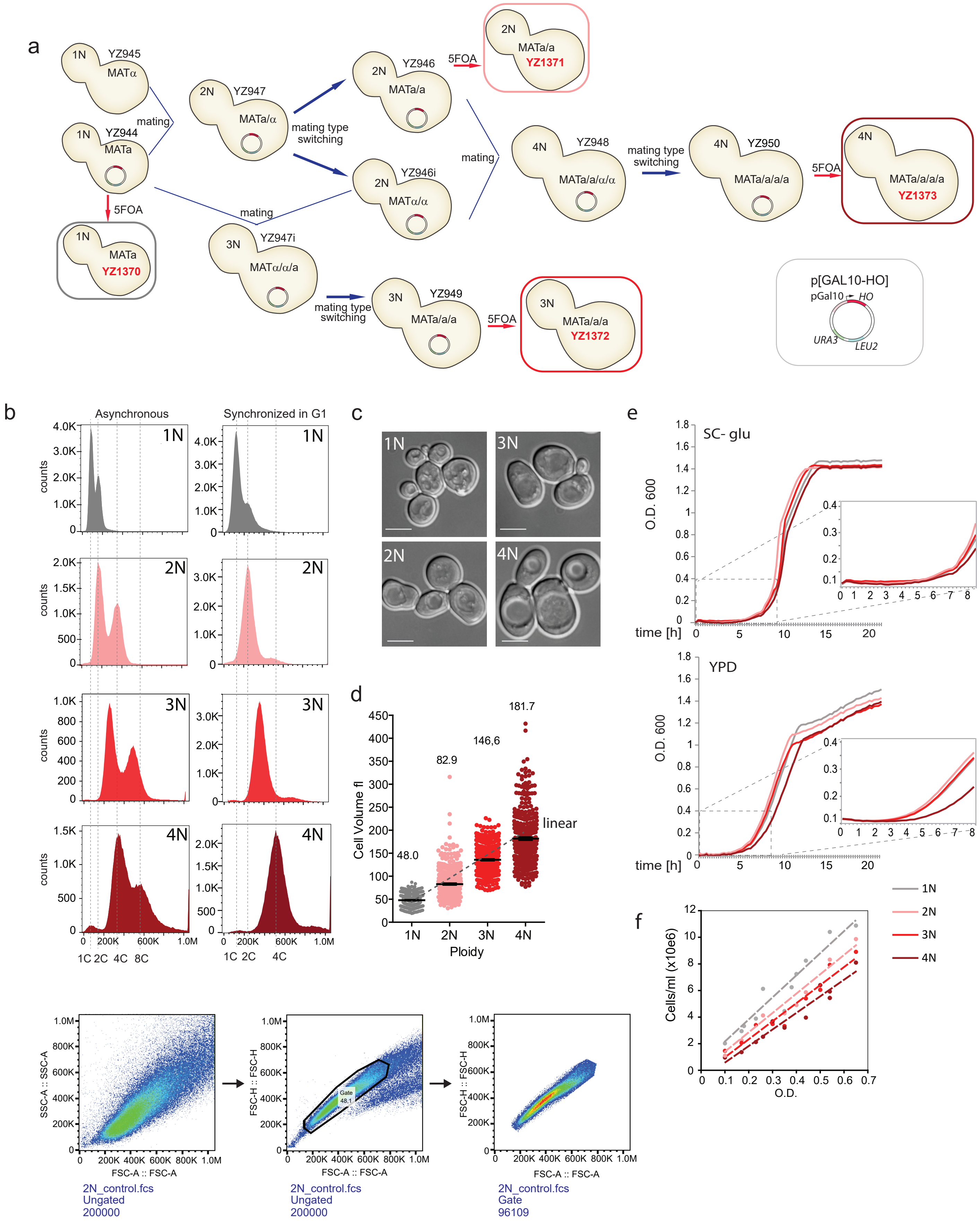
a
1N YZ945 MATα
1N YZ944 MATa
mating
2N YZ947 MATa/α
mating type switching
2N YZ946 MATa/a
2N YZ946i MATα/α
5FOA
2N MATa/a YZ1371
mating
4N YZ948 MATa/a/α/α
mating type switching
4N YZ950 MATa/a/a/a
5FOA
4N MATa/a/a/a YZ1373
5FOA
1N MATa YZ1370
mating
3N YZ947i MATα/α/a
mating type switching
3N YZ949 MATa/a/a
5FOA
3N MATa/a/a YZ1372
p[GAL10-HO]
pGal10
HO
URA3
LEU2
b
Asynchronous
Synchronized in G1
counts
1N
2N
3N
4N
1C 2C 4C 8C
1C 2C 4C
c
1N
3N
2N
4N
d
Cell Volume fl
48.0
82.9
146,6
181.7
linear
Ploidy
1N 2N 3N 4N
e
SC- glu
YPD
O.D. 600
time [h]
f
Cells/ml (x10e6)
O.D.
1N
2N
3N
4N
SSC-A :: SSC-A
FSC-A :: FSC-A
FSC-H :: FSC-H
Gate 48.1
2N_control.fcs
Ungated
200000
2N_control.fcs
Ungated
200000
2N_control.fcs
Gate
96109

Supplementary Figure 1

Cell volume and proliferation changes in cells of different ploidy

a Schematic depiction of the construction of isogenic yeast strains that differ only in their ploidy. The plasmid p[Gal10-HO] was used for the inducible mating type switching. The marked strains were used for the analysis. Further details can be found in Material and Methods. *Bottom right*: Schematics of the plasmid carrying the inducible HO endonuclease. **b** Flow cytometry analysis to determine the DNA content of the used strains. Bottom: The gating strategy used for the flow cytometry analysis.

c Representative images of yeast cells of different ploidy. Scale bar 5μm. **d** Quantification of the cell volume of yeast cells of different ploidy. 400 cells from asynchronous culturewere analyzed in four independent experiments; only small budded cells were included and the volume of the mother cell was measured. Dashed line marks linear scaling. *Top*: Mean volume. **e** Proliferation of yeast cells of different ploidy in fully supplemented synthetic medium with glucose as the carbone source (S°C-glu) and in a complete medium with glucose (YPD) at 30 C. **f** Calibration of optical density O.D. 600 and cell counts in cells of different ploidy. O.D. is affected by cell size and shape.

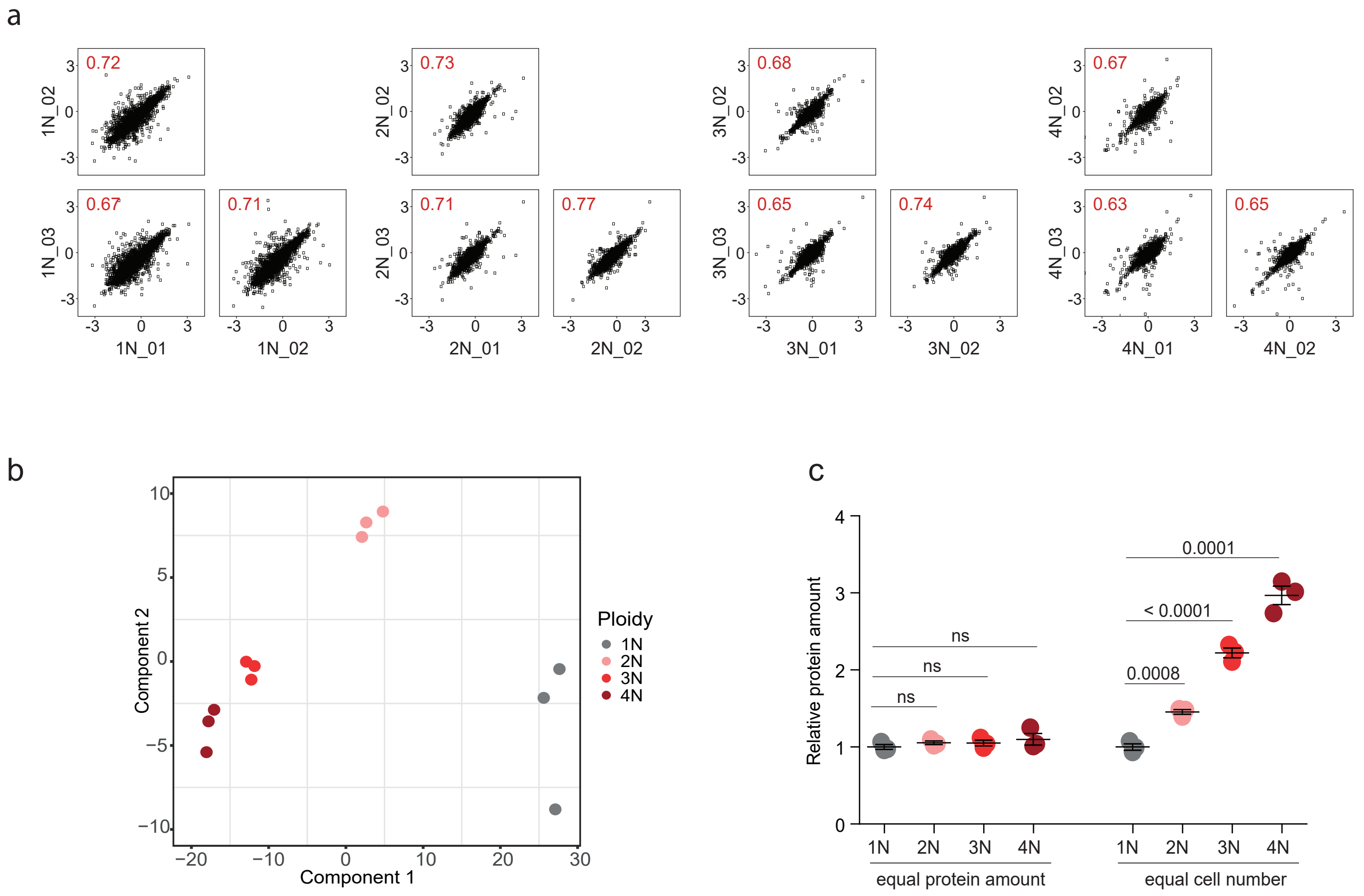

Supplementary Figure 2

Proteome analysis in cells of different ploidy

a Correlation of the normalized intensities of relative to those of the super SILAC standard. Individual biological replicates of each ploidy are plotted. **b** Principal component analysis of the individual MS/MS measurements. **c** Quantification of the protein levels from Fig. 1c. Three independent experiments were performed. The plots show means with SEM, the statistical evaluation was performed using the two-tailed, unpaired T test, p values are shown. ns - not significant. Source data are provided as a Source Data file.

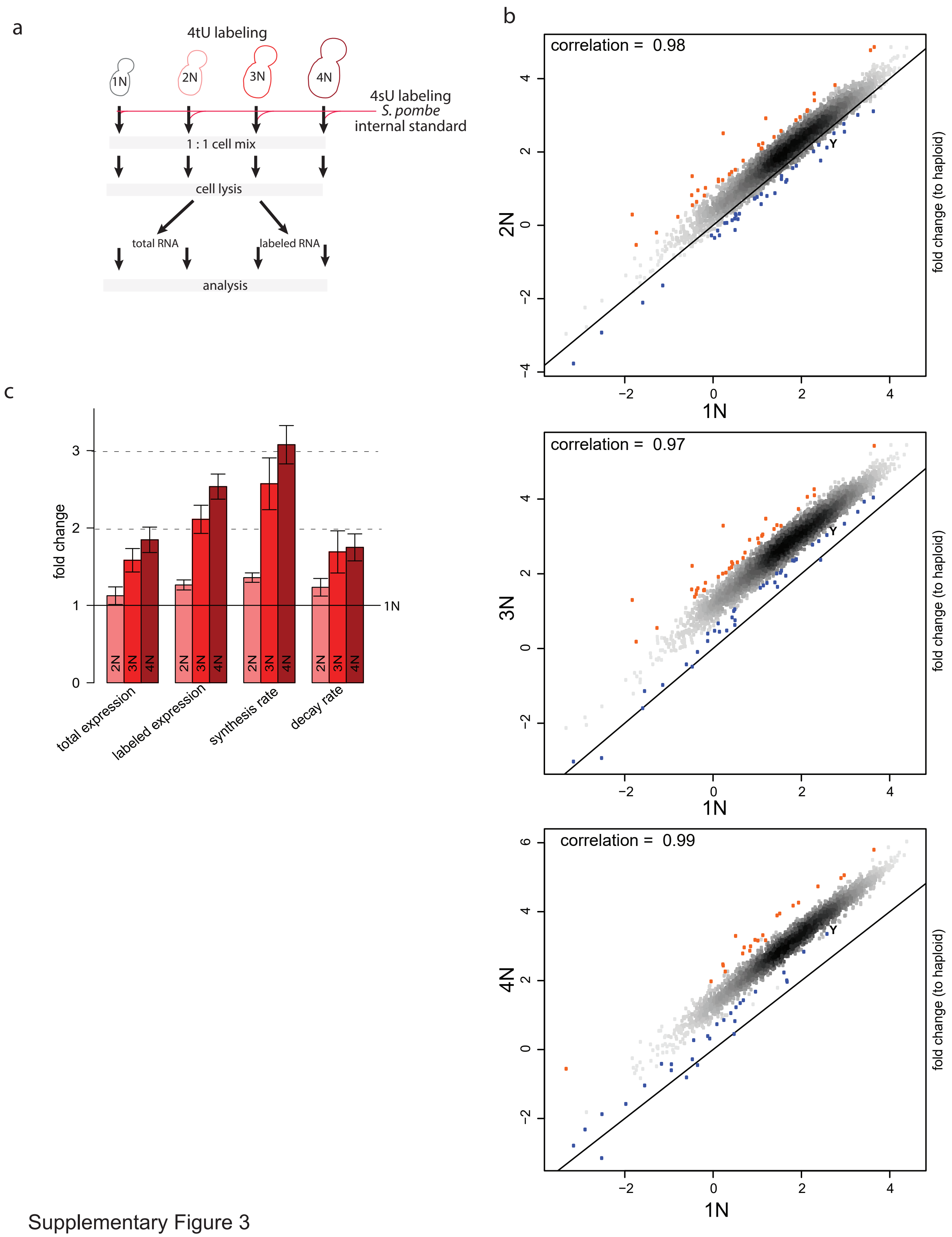

Supplementary Figure 3

Transcriptome changes in cell with altered ploidy

a Schematic depiction of the strategy used for transcriptome analysis. **b** mRNA abundance changes in strains of different ploidy compared to 1N strain. Relative transcriptome comparison. Red dots mark significantly upregulated, blue dots significantly downregulated mRNAs. Cut off 1.5 fold expression change. **c** Fold changes of total and labeled expression, synthesis and decay rate of mRNA in cells of different ploidy; normalized to haploid (1N) cells. Medians with confidence intervals of three independent replicates, >5500 values each, are shown.

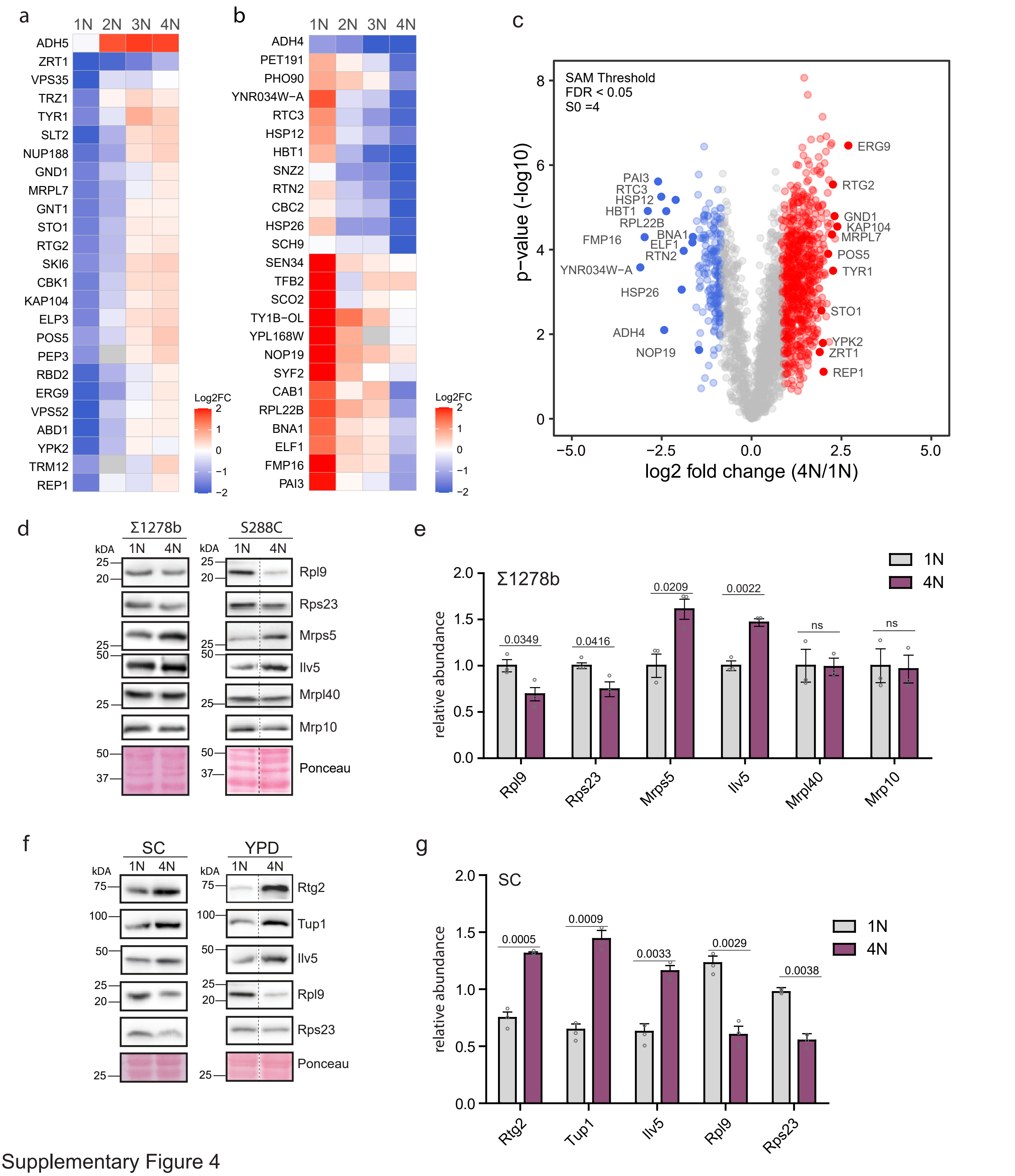

Supplementary Figure 4

Proteins differentially regulated with ploidy

a Top 25 proteins upregulated in tetraploids (4N) compared to haploids (1N). **b** Top 25 proteins downregulated in 4N compared to 1N. **c** Protein abundance changes in tetraploid cells relative to haploid cells. Log2 fold changes were plotted against the p-value (Student's T-test). Significantly deregulated proteins were identified using a permutation-based algorithm implemented in Perseus (FDR < 0.05, S0=4). Only the proteins with three valid values in each replicate were labeled(N=3). **d** Representative immunoblot of proteins in 1N and 4N cells derived from Σ1278b strains (*left*) and from S288C background. **e** Quantification of the protein levels in Σ1278b. Means and SEM of three independent experiments are shown. **f** Representative immunoblot of selected proteins in 1N and 4N cells grown in synthetic (SC, *left*) and rich (YPD) media. **g** Quantification of the immunobloting in **f**(SC medium). Means with SEM of three independent experiments. Statistical evaluation in **e**, **g**: Unpaired, two-tailed Student's t-test.

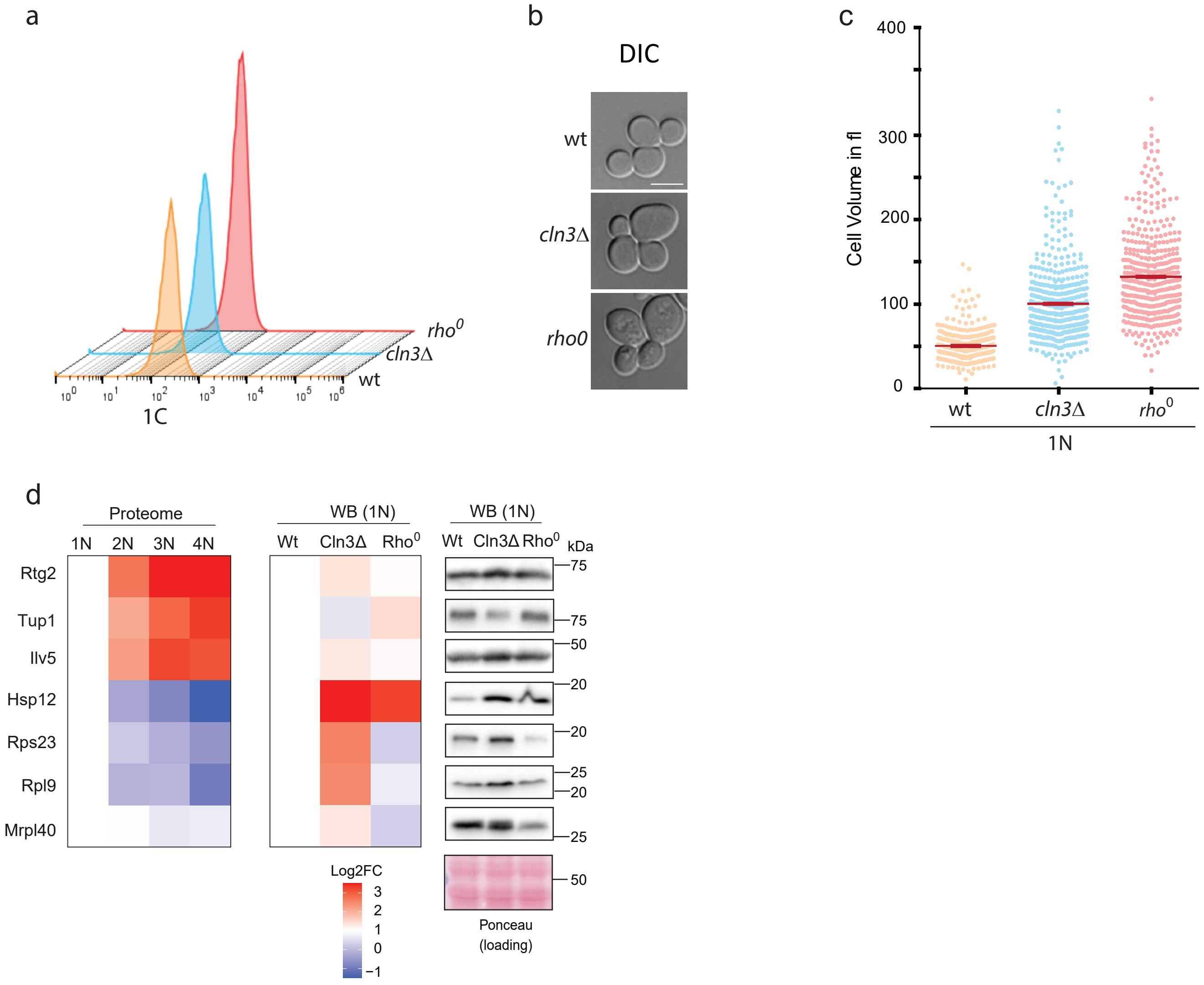

Supplementary Figure 5

Cell volume-dependent gene expression changes differ from changes due to increased ploidy

a Histogram of the DNA content in the mutant strains synchronized in G1 measured by flow cytometry. **b** Representative images of the analyzed yeast mutants. Scale bar 5 μm. **c** Quantification of the cell volume in used yeast mutants. Cell volume of >400 cells was calculated as indicated in the Materials and Methods. The average cell volumes of the indicated strains were 50.43, 100.3, and 132.4 fl, respectively. **d** Representative immunoblot and quantification of protein levels based on immunoblotting of whole cell lysates of the respective haploid mutant strains and their comparison to yeast strains of different ploidy.

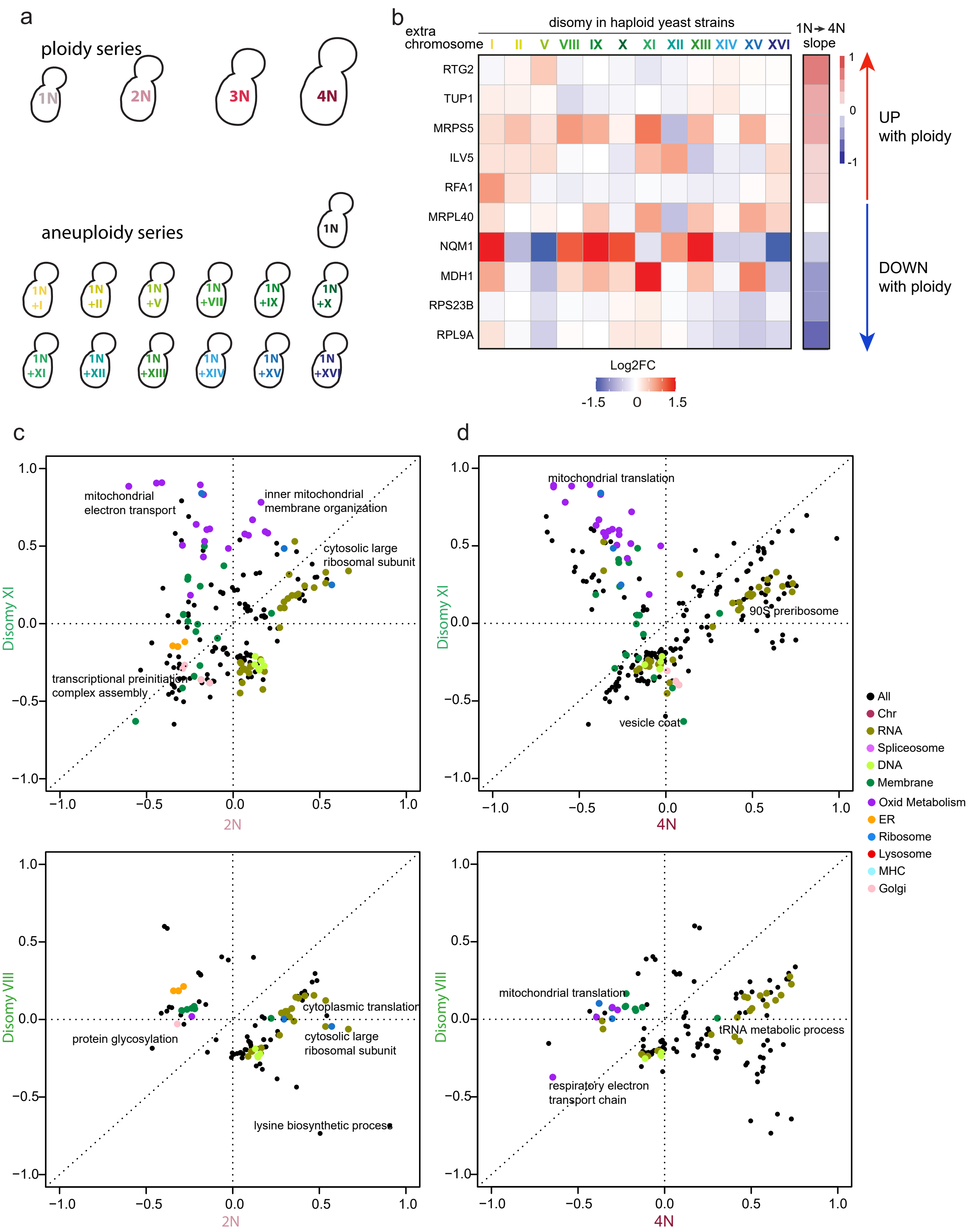

Supplementary Figure 6

Proteins differentially regulated in response to aneuploidy do not overlap with response to ploidy

a Schematic representation of compared strains: isogenic ploidy series was compared with isogenic disomy series, where haploid strains carried an extra copy of one specific chromosome. **b** Relative abundance changes of 10 proteins in disomic yeast strains normalized to isogenic haploid; *right*: protein abundance in strain of different ploidy. **c** Representative two-dimensional pathway annotation analysis comparing diploid and tetraploid strains (**d**) with disomic strains.

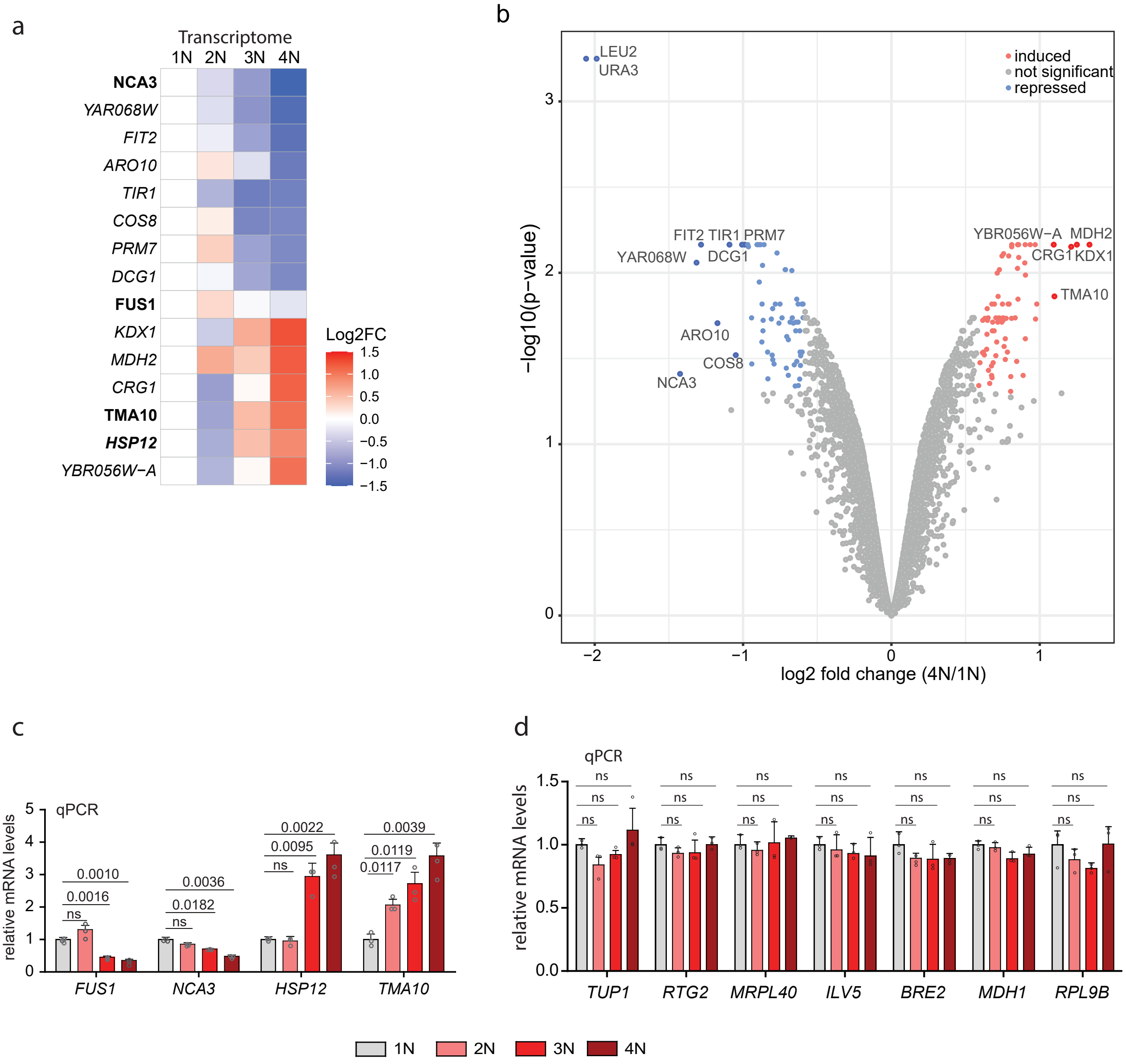

Supplementary Figure 7

Transcriptome changes in cell with altered ploidy

a Heat map of the most differentially regulated transcripts normalized to 1N. Transcripts marked in bold were validated by qRT-PCR shown in **c. b** Volcano plot of the relative abundance changes of mRNAs in 4N cells normalized ot 1N cells. *LEU2* and *URA3* were used as auxotrophic markers in this experiment. P values were calculated using the two-sided Student's t-test. **c** Quantification of mRNA levels of selected differentially regulated candidates in cells of different ploidy normalized to 1N. **d** Quantification of mRNA abundance by qRT-PCR of the candidates upregulated on protein level, normalized to 1N. Means and SD of three independent experiments are shown. Unpaired, two-tailed Student's t-test was used for statistic evaluation in **c** and **d**. Source data are in the Suplementary data file 2 (**a, b**) and in Source data file (**c,d**).

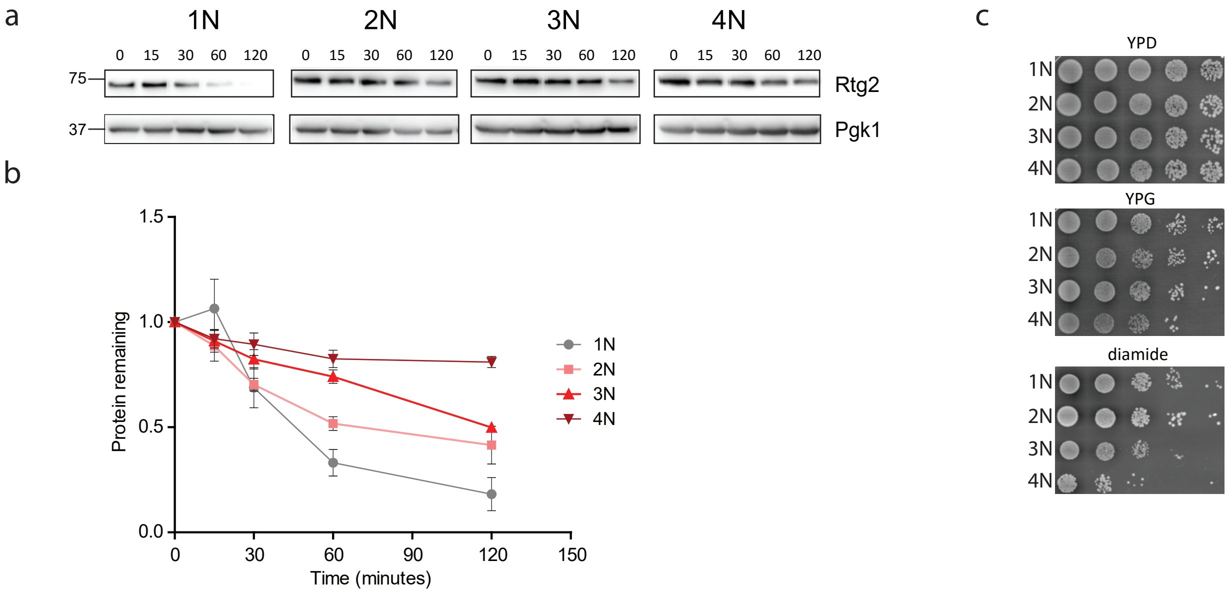

Supplementary Figure 8

Changes in mitochondrial metabolism in cells with higher ploidy

a Stabilization of Rtg2 with increasing ploidy. **b** Quantification of the plots in **a**. Means and SEM of three biological replicates are shown. **c** Reduced proliferation of isogenic yeast strains of different ploidy on non-fermentable medium and in presence of the oxidazing agent diamide.

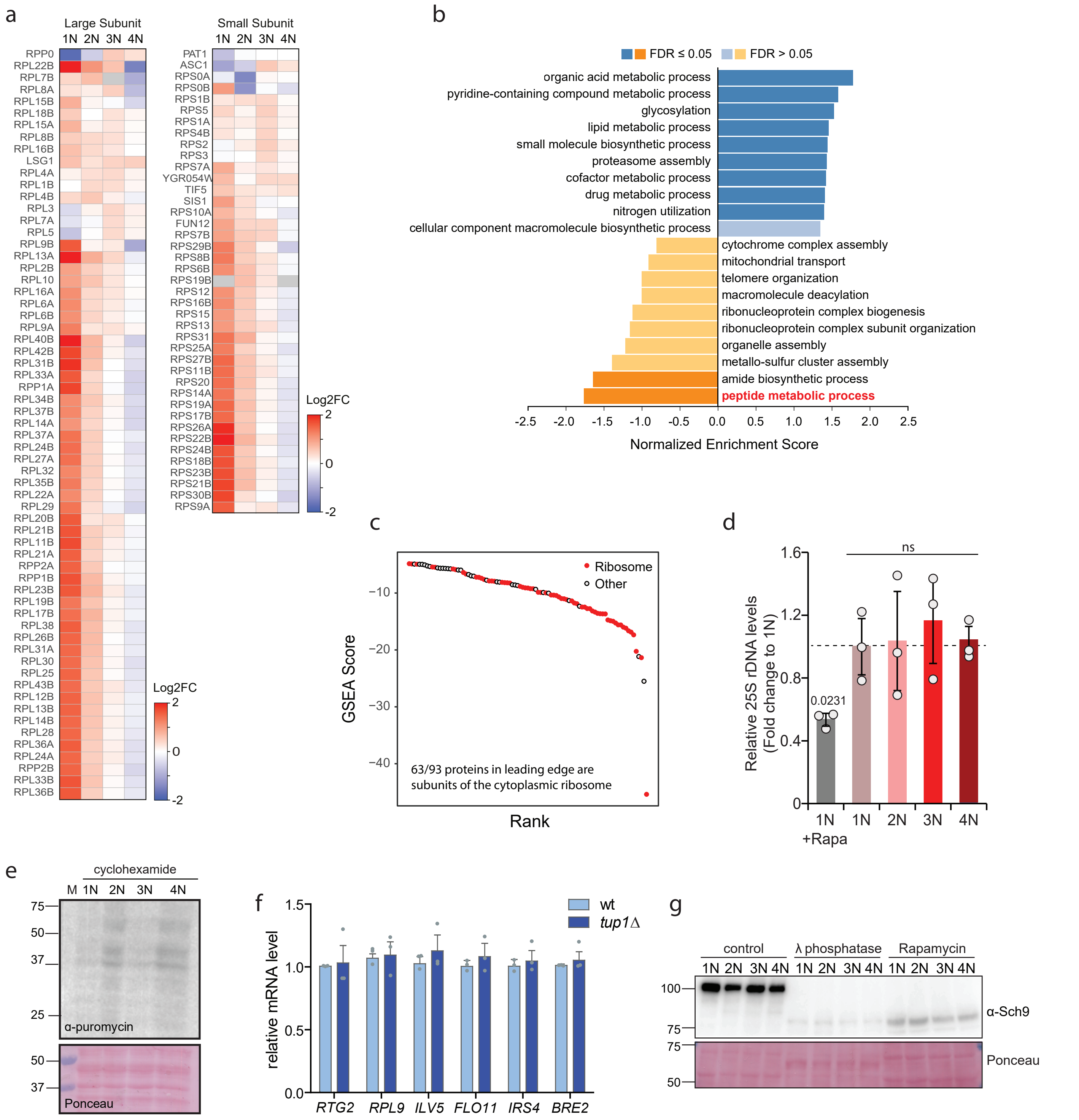

Supplementary Figure 9

Downregulation of the cytoplasmic ribosome in cells with increased ploidy

a Abundance of ribosomal proteins in cells of different ploidy. **b** Gene Set Enrichment Analysis carried out using the two-sided t-statistic derived from a linear model fitted to proteome L/H ratios for the different ploidy states. For details see Material and methods. **c** The WEBGESTALT score of each member of the GOBP category peptide metabolic process in the leading edge was plotted against the rank and ribosomal proteins were colored as indicated. **d** Relative abundance of 25S rDNA in cells of different ploidy. As a control, 1N cells were treated with rapamycin (Rapa) over prolonged time to reduce the rDNA copy number. Data present the mean of three independent replicates, error bars present standard deviation; unpaired two-tailed t-test was used. **e** Treatment with cyclohexamide abolishes puromycin incorporation. Control to Figure 2b. **f** Abundance of selected mRNAs in haploid wild type (WT) and tup1Δ strain quantified by rtPCR. Data present the mean of three independent replicates, error bars present standard deviation; unpaired two-tailed t-test was used. **g** Abundance of Sch9 and its phosphorylation in cells of different ploidy and upon rapamycin and phosphatase treatments. ns - not significant.

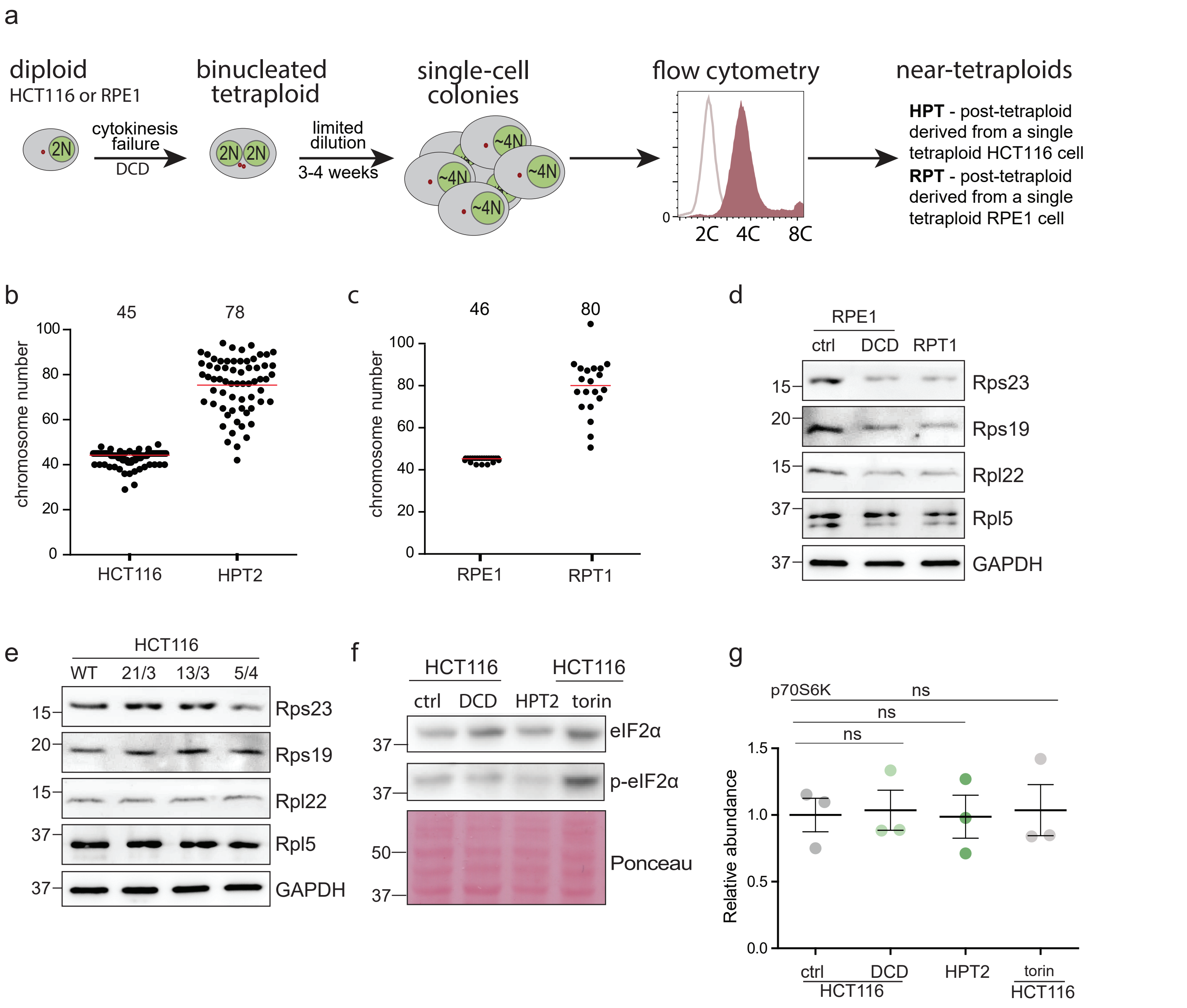

Supplementary Figure 10

Human tetraploid, near-tetraploid and aneuploid cell lines

a Schematic depiction of the construction of post-tetraploid cell lines. **b** Chromosome numbers in parental HPT116 and in posttetraploid HPT2 clone. Modal chromosome number is on the top of the plot and marked as a red line. n=63 cells in each sample were examined over 3 independent experiments. **c** Chromosome numbers in parental RPE1 and in posttetraploid RPT1 clone. Modal chromosome number is on the top of the plot and marked with a red line. n=22 cells each were examined over 2 independent experiments. **d** Representative immunoblot of ribosomal proteins in RPE1 and its tetraploid derivatives. **e** Representative immunoblot of ribosomal proteins in trisomic and tetrasomic cell lines engineered by microcell-mediated chromosome transfer in HCT116. 21/3 - trisomy of chromosome 21, 13/3 - trisomy of chromosome 16, 5/4 - tetrasomy of chromosome 5. **f** Representative immunoblot of eIF2a and P-eIF2a in diploid and tetraploid HCT116-derived cells. **g** Relative abundance of p70-S6K. Mean and SEM are shown in the plots; three independent experiments were performed. Unpaired, two-tailed Student's test was used for statistical evaluation.
