# Supplementary material for: Sublinear scaling of the cellular proteome with ploidy
Source: Nat Commun. 2022 Oct 19;13:6182. doi: 10.1038/s41467-022-33904-7 (PMC9581932; doi:10.1038/s41467-022-33904-7)
Supplement: Supplementary file 6 — Reporting Summary [file 41467_2022_33904_MOESM6_ESM.pdf]

nature portfolio

Corresponding author(s): Zuzana Storchova

Last updated by author(s): 2022 07 13

Reporting Summary

Nature Portfolio wishes to improve the reproducibility of the work that we publish. This form provides structure for consistency and transparency in reporting. For further information on Nature Portfolio policies, see our Editorial Policies and the Editorial Policy Checklist.

Statistics

For all statistical analyses, confirm that the following items are present in the figure legend, table legend, main text, or Methods section.

n/a	Confirmed	
☐	☒	The exact sample size (*n*) for each experimental group/condition, given as a discrete number and unit of measurement
☐	☒	A statement on whether measurements were taken from distinct samples or whether the same sample was measured repeatedly
☐	☒	The statistical test(s) used AND whether they are one- or two-sided<br>*Only common tests should be described solely by name; describe more complex techniques in the Methods section.*
☒	☐	A description of all covariates tested
☐	☒	A description of any assumptions or corrections, such as tests of normality and adjustment for multiple comparisons
☐	☒	A full description of the statistical parameters including central tendency (e.g. means) or other basic estimates (e.g. regression coefficient) AND variation (e.g. standard deviation) or associated estimates of uncertainty (e.g. confidence intervals)
☐	☒	For null hypothesis testing, the test statistic (e.g. *F*, *t*, *r*) with confidence intervals, effect sizes, degrees of freedom and *P* value noted<br>*Give P values as exact values whenever suitable.*
☒	☐	For Bayesian analysis, information on the choice of priors and Markov chain Monte Carlo settings
☐	☒	For hierarchical and complex designs, identification of the appropriate level for tests and full reporting of outcomes
☐	☒	Estimates of effect sizes (e.g. Cohen's *d*, Pearson's *r*), indicating how they were calculated

Our web collection on statistics for biologists contains articles on many of the points above.

Software and code

Policy information about availability of computer code

Data collection	Western blots were imaged using Azure c500<br>Promega Glomax Explorer microplate reader for measuring luminescence (Cell titer glo based assays)<br>Attune NxT Flow Cytometer (Thermo Fisher Scientific)<br>DIC and immunofluorescence images were captured using AxioObserver Z1 equipped with CSU-X1 spinning disk confocal head (Yokogawa) and Laser stack launch (3I, Denver, CO)<br>Mass spectrometry data were obtained with Q Exactive mass spectrometer (Thermo Fisher Scientific)<br>Dynamic transcription analysis was performed using GeneChip Yeast Genome 2.0 microarrays (Affymetrix).
Data analysis	GraphPad Prism 5,MaxQuant v1.6.3.3,Perseus v1.6.8, R v3.6.1,R package: ggplot2 v3.2.0,R package: dplyr v0.8.3<br>R package: reshape2 v1.4.3,R package: gtools v 3.8.1,R package: EQL v1.0-1,R package: kdensity v1.0.1,R package:<br>Limma v3.42.2,R package:VSN v 3.54.0,R/Bioconductor, DTA package, Slidebook 6, BudJ, ImageJ 1.53, FlowJo/LLC, Attune™ Cytometric Software 3.1.2, Webgestalt 2019

For manuscripts utilizing custom algorithms or software that are central to the research but not yet described in published literature, software must be made available to editors and reviewers. We strongly encourage code deposition in a community repository (e.g. GitHub). See the Nature Portfolio guidelines for submitting code & software for further information.

Data

Policy information about availability of data

All manuscripts must include a data availability statement. This statement should provide the following information, where applicable:

- Accession codes, unique identifiers, or web links for publicly available datasets
- A description of any restrictions on data availability
- For clinical datasets or third party data, please ensure that the statement adheres to our policy

Transcriptome data have been deposited in the Gene Expression Omnibus database under accession code GSE162513.
The mass spectrometry proteomics data have been deposited to the ProteomeXchange Consortium via the PRIDE partner repository with the dataset identifier PXD022605.
Normalized data are available in the Supplementary datasets and can be visualized through a web-based application PloiDEx.
The Uniprot FASTA database UniProt ID: UP000002311 was used to search for yeast peptides.

Human research participants

Policy information about studies involving human research participants and Sex and Gender in Research.

Reporting on sex and gender	n/a
Population characteristics	n/a
Recruitment	n/a
Ethics oversight	n/a

Note that full information on the approval of the study protocol must also be provided in the manuscript.

Field-specific reporting

Please select the one below that is the best fit for your research. If you are not sure, read the appropriate sections before making your selection.

[x] Life sciences [ ] Behavioural & social sciences [ ] Ecological, evolutionary & environmental sciences

For a reference copy of the document with all sections, see nature.com/documents/nr-reporting-summary-flat.pdf

Life sciences study design

All studies must disclose on these points even when the disclosure is negative.

Sample size	A minimum of 3 biological replicates (note that in each replicate sample, a large population of cells (from several hundreds to 10e8) was analyzed) were performed. N is specified in figure legends.
Data exclusions	No data were excluded from the analysis
Replication	All the experiments were performed from at least 3 independent experiments (unless stated otherwise), the reproducibility was statistically evaluated, the applied statistical tests are noted in figure legends or in material and methods. Three biological replicates are the minimal number to allow statistical analysis and are standardly used in these types of assays.
Randomization	Randomization was not required, because the analyzed populations are extremely large (10e8 cells) and therefore confounding effect will likely not influence the results. Moreover, all experiments were performed in at least three biological replicates.
Blinding	Blinding was used for the data image analysis (Supplementary Figure 1d, f) - the sample names were altered.

Reporting for specific materials, systems and methods

We require information from authors about some types of materials, experimental systems and methods used in many studies. Here, indicate whether each material, system or method listed is relevant to your study. If you are not sure if a list item applies to your research, read the appropriate section before selecting a response.

Materials & experimental systems

n/a	Involved in the study
☐	☒ Antibodies
☐	☒ Eukaryotic cell lines
☒	☐ Palaeontology and archaeology
☒	☐ Animals and other organisms
☒	☐ Clinical data
☒	☐ Dual use research of concern

Methods

n/a	Involved in the study
☒	☐ ChIP-seq
☐	☒ Flow cytometry
☒	☐ MRI-based neuroimaging

Antibodies

Antibodies used

Mrp10 Rabbit Gift from Prof. Dr. Johannes M Herrmann
Mrpl40 Rabbit Gift from Prof. Dr. Johannes M Herrmann
Oxa1 Rabbit Gift from Prof. Dr. Johannes M Herrmann
Ilv5 Rabbit Gift from Prof. Dr. Johannes M Herrmann
Mrps5 Rabbit Gift from Prof. Dr. Johannes M Herrmann
Mdh1 Rabbit Gift from Prof. Dr. Johannes M Herrmann
Clb2 Sc-9071 Rabbit Santa Cruz - recommended for detection of Clb2 of Saccharomyces cerevisiae origin by Western Blotting
Rps23B Sc-100837 mouse Santa Cruz - recommended for detection of Ribosomal Protein S23 of mouse, rat and human origin by WB, IP, IF and ELISA
Rpl9 AP16409b-ev Rabbit ABGENT
Sch9 ab56203 Rabbit Abcam - Rabbit polyclonal suitable for ELISA, WB
PAP P1291 Rabbit Sigma -Aldrich - used for all TAP-tagged proteins
Anti-HA-Tag (F-7) Sc-7392 mouse Santa Cruz -HA-Tag Antibody (F-7) is recommended for detection of proteins containing the HA tag by WB, IP, IF, FCM and ELISA
Anti-Puromycin (clone 12D10) MABE343 mouse Merck - demonstrated to react with Human test sample, preincubated with Puromycin. Predicted to react with all species when test sample is incubated with Puromycin.
Rps21 16946-1-AP Rabbit Proteintech - targets RPS21 in WB, IHC, IF,ELISA applications and shows reactivity with human, rat, mouse samples
Rps8 Ab201454 Rabbit Abcam - Validated in WB, IP, ICC/IF, Flow Cyt (Intra) and tested in Mouse, Rat, Human.
Rps19 Sc-100836 mouse Santa Cruz - a monoclonal antibody recommended for WB, IP, IF and ELISA.
Rpl22 Sc-136413 mouse Santa Cruz - recommended for detection of Ribosomal Protein L22 of mouse, rat, human and canine origin by WB and IP
Rpl5 Ab86863 Rabbit Abcam - Suitable for: IP, IHC-P, WB, ICC/IF, recognizes human Rpl5
Rpl21 Sc-393663 mouse Santa Cruz - is recommended for detection of Ribosomal Protein L21 of mouse, rat and human origin and Ribosomal Protein L21-like of mouse and rat origin by WB, IP, IF and ELISA
Tle1 sc-137098 mouse Santa Cruz - is mouse monoclonal IgG1 κ TLE1 antibody, recommended for detection of TLE1 of mouse, rat and human origin by WB, IP, IF and ELISA
GAPDH (14C10) 2118 Rabbit Cell signaling - detects endogenous levels of total GAPDH protein in Human, Mouse, Rat, Monkey, Bovine, Pig WB
p70 S6 Kinase (49D7) 2708 Rabbit Cell signaling - monoclonal antibody fvalidated for Western Blotting
Phospho-p70 S6 Kinase (Thr389) 9205 Rabbit Cell signaling - polyclonal antibody, validated for Western Blotting
Secondary antibody
Goat anti-rabbit HRP HAF008 R&D
Goat antimouse HRP HAF007 R&D
Supplementary table 2 lists all antibodies used in this study.

Validation

For western blot and immunofluorescence, all commerically available antibodies were used according to the validations performed by the manufacturers. Custom made antibodies were validated in yeast strains by analysis of cell extracts and isolated mitochondria derived from deletion mutants the laboratory of the donor, Prof. Dr. Johannes Herrmann. For reference see PMID: 24360785, PMID: 14657018 and PMID: 34786732.

Eukaryotic cell lines

Policy information about cell lines and Sex and Gender in Research

Cell line source(s)

HCT116 (45,X) were purchased from ATCC® (CCL-247). hTERT RPE-1 cells (46,XX) were purchased from ATCC® (CRL-4000). Whole genome doubling was induced by treatment with cytochalasin D as described previously. Sequencing and karyotyping was performed previously and is described in Kuznetsova et al, Cell Cycle 2015 (referenced).

Authentication

No authentication as such was performed. However, structural aneploidies typical for HCT116 and RPE1 (e.g. gain of chromosome 10q, a known karyotype characteristic of RPE1 cells) was determined. This finding was validated using whole genome sequencing and spectral karyotyping (Kuznetsova et al, Cell Cycle 2015 - referenced in the paper).

Mycoplasma contamination

All the cell lines tested negative for mycoplasma contamination using PlasmoTest (InvivoGen).

Commonly misidentified lines (See ICLAC register)	No commonly misidentified cell lines were used in this study.

Flow Cytometry

Plots

Confirm that:

- [x] The axis labels state the marker and fluorochrome used (e.g. CD4-FITC).
- [x] The axis scales are clearly visible. Include numbers along axes only for bottom left plot of group (a 'group' is an analysis of identical markers).
- [x] All plots are contour plots with outliers or pseudocolor plots.
- [x] A numerical value for number of cells or percentage (with statistics) is provided.

Methodology

Sample preparation	Yeast strains and human cell lines were labelled with propidium iodide. Cells were fixed and permeabilized for 15 min with Fix perm (Thermo Fisher scientific), and resuspended in PBS containing RNase (10 µg/mL) and PI.
Instrument	Attune NxT acoustic focusing flow cytometer
Software	Attune NxT Software 3.1.1243.0
Cell population abundance	Total cells:>80%, Single cells: > 90%
Gating strategy	no gating was used

- [x] Tick this box to confirm that a figure exemplifying the gating strategy is provided in the Supplementary Information.
